# Anti-onchocerca Metabolites from *Cyperus articulatus*: Isolation, In Vitro Activity and In Silico ‘Drug-Likeness’

**DOI:** 10.1007/s13659-014-0023-5

**Published:** 2014-06-10

**Authors:** Jonathan Alunge Metuge, Smith B. Babiaka, James A. Mbah, Fidele Ntie-Kang, Godfred A. Ayimele, Fidelis Cho-Ngwa

**Affiliations:** 1 ANDI Centre of Excellence, Department of Biochemistry and Molecular Biology, Faculty of Science, University of Buea, P. O. Box 63, Buea, Cameroon; 2 Department of Chemistry, Faculty of Science, University of Buea, P. O. Box 63, Buea, Cameroon; 3 Chemical and Bioactivity Information Centre, Chemistry Department, Faculty of Science, University of Buea, P. O. Box 63, Buea, Cameroon

**Keywords:** *Cyperus articulates*, Linoleic acid, Microfilariae, Mustakone, *Onchocerca ochengi*, *Onchocerca volvulus*

## Abstract

The aims of this investigation were to isolate active ingredients from the roots/rhizomes of *Cyperus articulatus* used as herbal medicine in Cameroon for the treatment of human onchocerciasis and to assess the efficacy of the metabolites on the *Onchocerca* worm. The antifilarial activity was evaluated in vitro on microfilariae (Mfs) and adult worms of the bovine derived *Onchocerca ochengi*, a close relative of *Onchocerca volvulus*. Cytotoxicity was assessed in vitro on monkey kidney epithelial cells. The structures of the active compounds were determined using spectroscopic methods and their drug-likeness evaluated using Lipinski parameters. Two secondary metabolites, AMJ1 [containing mustakone (**1**) as the major component] and linoleic acid or (9*Z*,12*Z*)-octadeca-9,12-dienoic acid (**2**) were isolated. Both compounds were found to kill both the microfilariae and adult worms of *O. ochengi* in a dose dependent manner. The IC_50_s for AMJ1 were 15.7 µg/mL for Mfs, 17.4 µg/mL for adult males and 21.9 µg/mL for adult female worms while for linoleic acid the values were, 15.7 µg/mL for Mfs, 31.0 µg/mL for adult males and 44.2 µg/mL for adult females. The present report provides the first ever evidence of the anti-*Onchocerca* efficacy of AMJ1 and linoleic acid. Thus, these secondary metabolites may provide a lead for design and development of new antifilarial agents.

**Electronic supplementary material:**

The online version of this article (doi:10.1007/s13659-014-0023-5) contains supplementary material, which is available to authorized users.

## Introduction

Human onchocerciasis or subcutaneous filariasis is a parasitic disease caused by the filarial worm *Onchocerca volvulus*. It is transmitted through the bites of infected blackflies of *Simulium* species, which carry immature larval forms of the parasite from human to human. In the human body, the larvae form nodules in the subcutaneous tissue, where they mature to adult worms. After mating, the female adult worm can release up to 1000 microfilariae a day. These move through the body, and when they die they cause a variety of conditions, including blindness, skin rashes, lesions, intense itching and skin depigmentation [1]. About 37 million persons are infected with *O. volvulus*, of whom 270000 are blind and 500000 visually impaired with about 90 million vulnerable to get the disease [2, 3]. The burden of onchocerciasis causes long term disability, social stigmatization and leads to a highly unproductive population and consequently to economic loss and slowdown of country development over the years since the affected population is forced to abandon the infested areas which usually have a high agricultural potential [4].

Ivermectin (IVM), the sole drug used in community-directed treatment of onchocerciasis is microfilaricidal and with only limited activity against the adult worms. Parasite resistance to IVM and adverse reactions observed in patients co-infected with *Loa loa* limit the use of the drug [3]. To date, the only known macrofilaricide for onchocerciasis is suramin but it is toxic. There is need to develop safe and easily administered drugs that can kill the adult *O. volvulus* parasite to reduce the time needed for control programs to eliminate adult worms from an endemic area. Rational drug discovery approaches have made only limited advances in the discovery of a safe macrofilaricide against the *Onchocerca* worm. It has been suggested that plants used in folk medicine to treat parasitic diseases may provide an alternative source of macrofilaricides [5]. Previous studies in our laboratory showed that the hexane extract of the roots/rhizomes of *Cyperus articulatus* (used as herbal medicine in Cameroon to treat onchocerciasis) was active against the microfilariae and adult worms of the bovine parasite *Onchocerca ochengi*, a close relative of the medically important *O. volvulus*. *C. articulatus* (family: Cyperaceae) is a bushy grass mainly found along tropical rivers and streams. The present studies aimed at isolating pure compounds from the hexane extract (essential oil) of the roots/rhizomes of the plant and evaluate their anti-*Onchocerca* activity and drug-likeness.

## Results

### Identification of Secondary Metabolites Isolated from the Hexane Extract of *Cyperus articulatus*

**Fig. 1:**
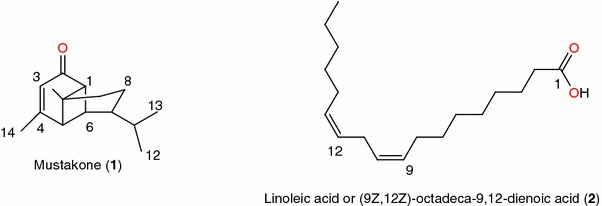
Structures of the compounds reported in this paper

The roots/rhizomes of *C. articulatus* were extracted with hexane. Column chromatography of this extract as described in the Experimental section afforded two compounds (Fig. 1) which were identified with the help of ^1^H and ^13^C NMR spectra as well as by comparison of these data with published literature values for mustakone [6, 7] and linoleic acid [8].

### Identification of AMJ1

AMJ1 was obtained as white oil. The ESI-MS (ESM Fig. S1) of AMJ1 revealed the presence of three compounds with a major compound with peak at 219.1. In fact, its ^1^H NMR spectrum showed a peak at 5.71 ppm characteristic of an olefinic proton deshielded by inductive effect of a carbonyl group. Other protons resonated at *δ* 1.50–1.70 ppm corresponding to axial and equatorial protons of a cyclohexane ring. The ^13^C NMR spectrum (ESM Table S1) showed the signals of the 15 carbon atoms amongst others. Comparing the ^1^H and ^13^C NMR data of mustakone (**1**) [6, 7] with those of AMJ1, further confirmed the structure, mustakone which was previously isolated from the rhizomes of *C. articulatus* [7] was the major compound.

**Fig. 2:**
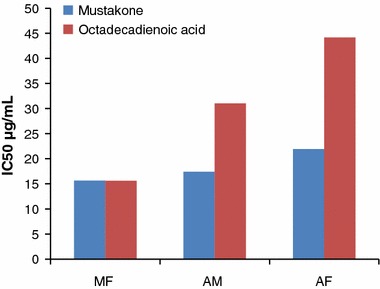
Effect of compounds from *C. articulatus* essential oil (hexane extract) on *O. ochengi* microfilariae (MF), adult male (AM) and adult female (AF) worms after 20 h incubation. The lower the IC_50_ value the more effective the compound

**Table 1:** IC_50_, IC_100_ and selectivity indices (SI) of mustakone and octadeca-9,12-dienoic acid on *O. ochengi* microfilariae and adult worms, and monkey kidney epithelial cells (LLC-MK2) in secondary screens

	AMJ1-containing mustakone				Linoleic acid			
	Microfilariae	Adult male worm	Adult female worm	Monkey kidney cells (LLC-MK2)	Microfilariae	Adult male worm	Adult female worm	Monkey kidney cells (LLC-MK2)
IC_50_ (µg/mL)	15.65	17.41	21.89	93.7	15.62	31.03	44.16	125
IC_100_ (μg/mL)	31.25	62.5	62.5		62.5	62.5	125	
SI	5.98	5.38	4.28		8.0	4.02	2.83	

### Identification of AMJ2

AMJ2 was obtained as white oil. Its ^1^H NMR spectrum showed signals characteristic of olefinic protons at *δ* 5.34–5.40, a methylene protons at *δ* 2.79 sandwiched between two *sp*^2^ carbon atoms and a methylene proton next to a carbon bearing an acid function at *δ* 2.34 (ESM Table S2). Other protons resonated at *δ* 2.00–1.25 corresponding to methylene protons and methyl protons up field at *δ* 0.88. Its ^13^C NMR spectrum showed 18 carbon atoms and revealed the presence of a carbonyl signal at *δ* 180.4 (C-1). The other signals were in close agreement with literature values [8]. Comparing these ^1^H NMR and ^13^C NMR data with those of linoleic acid [8] further confirmed the structure of AMJ2 to be linoleic acid (**2**) which was previously isolated from *Hydrangea chinensis* [8].

**Table 2:** Effect of compounds from roots/rhizomes of *C. articulatus* on viability of *O. ochengi* microfilariae (Mfs) and adult worms at 120 h of culturing parasites in primary screens

Test substance^a^	% Microfilarial motility reduction	% Adult male worm motility reduction	% Adult female worm death	Comment
AMJ1 (500 μg/mL)	100	100	100	Macro- and microfilaricidal
Linoleic acid (500 μg/mL)	100	100	100	Macro- and microfilaricidal
Ivermectin (10 μg/mL)	100	NA	NA	Microfilaricidal
(10 µM NYBC01)	100	100	100	Macro- and microfilaricidal
2 % DMSO	0	0	0	Inactive

*NA* not active

^a^The gold-conjugated compound, NYBC01 was used as positive control for adult worm assay while ivermectin which is known not to kill adult worms was used for Mf assay. Dimethyl sulphoxide (DMSO) was used as negative control. Percentage adult female worm death corresponds to percentage inhibition of formazan formation

### Effect of Isolated Metabolites on *O. ochengi* Microfilariae and Adult Worms, and Monkey Kidney Epithelial Cells (LLC-MK2) in Secondary Screens

The concentration that causes 50 % growth inhibition (IC_50_) in *O. ochengi* microfilariae and adult worms, that which causes 100 % inhibition (IC_100_) as well as the selectivity indices against monkey kidney epithelial cells (LLC-MK2) in secondary screens are shown in Table 1, meanwhile Table 2 shows the effects of isolated secondary metabolites (AMJ1 and linoleic acid) on *O. ochengi* microfilariae and adult worms, and monkey kidney epithelial cells (LLC-MK2) in secondary screens. AMJ1 is more active on adult worms (lower IC_50_ values) than linoleic acid (Table 2; Fig. 2). Both metabolites exhibit greater activity on adult male than on adult female worms. A comparison of the effects of the two isolated metabolites on *O. ochengi* microfilariae (MF), adult male (AM) and adult female (AF) worms after 20 h incubation is shown in the histograms on Fig. 2.

For microfilariae (Mfs) and adult male worms IC_50_ corresponds to the concentration of the phytochemical that inhibits worm motility by 50 % while for adult female worms, IC_50_ is the concentration of the metabolite that inhibits formazan formation by 50 %. IC_50_ for mammalian cells (monkey kidney cells) is the concentration of the metabolite that inhibits cell viability by 50 %. SI (Selectivity Index) = IC_50_ on mammalian cells/IC_50_ on parasite. IC_100_ is the concentration of the bioactive metabolite that inhibits *O. ochengi* Mfs and adult male worm motility or formazan formation by adult female worms by 100 %.

### In Silico “Drug-likeness” Profile of Plant-Derived Compounds with Anti-onchocerca Activity

Lipinski et al. [9] postulated the “rule of five”, which states that a compound will likely not be orally bioavailable if its molecular weight (MW) is >500 Daltons (Da), its *n*-octanol/water partition coefficient (log *P*) >5, its number of H-bond acceptors (HBA) >10 and its number of H-bond donors (HBD) >5. An additional rule for the number of rotatable bonds (NRB) is often added to Lipinski’s rule in order to take drug metabolism and pharmacokinetics into account, since this parameter is known to influence bioavailability in rats [10]. The above parameters were used to initially access the likely oral bioavailability of the isolated compounds. Lipinski evaluation showed that whereas linoleic acid shows only one violation of the Lipinski rule, mustakone had no violation (Table 3).

**Table 3:** Summary of Lipinski parameters for the isolated compounds

Compound name	Log P	NRB	MW (Da)	HBA	HBD	Lipinski violations
Mustakone	2.82	1	218.34	1	0	0
Linoleic acid	5.48	14	280.45	2	2	1

## Discussion

Onchocerciasis causes intense itching, blurred vision and sometimes irreversible blindness. Rational drug discovery approaches have made only limited advances in the discovery of a safe macrofilaricide against the *Onchocerca* worm [11]. It has been suggested that medicinal plants used in folk medicine to treat parasitic diseases may provide an alternative source of macrofilaricides [5]. We here report the efficacy of AMJ1 [containing mustakone (**1**) as major component] and linoleic acid [(9*Z*,12*Z*)-octadeca-9,12-dienoic acid] (**2**) isolated from the essential oil (hexane extract) of the roots/rhizomes of *C. articulatus* against microfilariae and adult *O. ochengi* worms, the closest relative and best model of the human parasite, *O. volvulus* [11]. Thus, it is feasible that compounds that are active against the bovine parasite will also affect the human parasite, *O. volvulus*. The effect of these metabolites on the *Onchocerca* worm seems to be specific since other compounds tested under similar conditions were ineffective (results not shown). AMJ1-containing mustakone was more active in vitro than linoleic acid on adult male and female worms. The combined effect of these two metabolites may explain in part the apparent anti-*Onchocerca* activity of decoctions of *C. articulatus* and justifies the use of this plant as traditional medicine for the treatment of human onchocerciasis. However due to the difficulty in obtaining very pure compounds from oils, it may be necessary to further evaluate the anti-onchocerca activity of these compounds by using commercially produced samples. Furthermore HPLC techniques should be used to separate AMJ1 to determine the active principle in it. Mustakone, a sesquiterpene previously isolated by Nyasse et al. [6, 7] has been shown to exhibit anti-plasmodial activity [12]. Linoleic acid has also been reported to have anti-bacterial [13] and antifungal activity [14]. We here report for the first time their activity against the *Onchocerca* worm. Lipinski evaluation shows that these compounds are potential drug leads against onchocerciasis.

## Experimental Section

### General Experimental Procedures

^1^H and ^13^C NMR spectra were recorded at 500 and 125 MHz, respectively, on a Bruker ARX- 500 spectrometer. The ^1^H and ^13^C NMR chemical shifts are expressed in ppm relative to TMS. Chemical shifts were recorded in *δ* (ppm) and the coupling constants (*J*) are in hertz. Thin-layer chromatography (TLC) was performed on Merck silica gel plates. TLC plates were visualized with a UV-lamp (UVGL-58) at 254 or 366 nm and later exposed to iodine. Column chromatography was performed with glass columns using either silica gel 60–200 mesh or Sephadex LH-20. The relative molecular mass was obtained from Electron Spray Ionisation Mass Spectroscopy (ESI-MS) in the positive mode (Water). The analysis was obtained using a Nucleodur C18 Pyramid column (Macherey_Nagel).

### Isolation of Pure Compounds from the Hexane Extract of the Roots/Rhizomes of *Cyperus articulatus*

#### Plant Material

*Cyperus articulatus* was collected from inland valleys at Sehn village, Ndu Sub-Division in the North West Region of Cameroon, based on ethno pharmacological information. The local name of the plant is “Ndfu”. The voucher specimen was deposited at the National herbarium in Yaoundé and assigned voucher number 19450/SRF-CAM. The roots/rhizomes of the plant were separated from the stem, air dried and ground to fine powder.

#### Extraction and Isolation of Metabolites

The powdered (roots/rhizome) sample (700 g) was macerated in hexane (3 × 3 days each), filtered and concentrated on a rotavapor to dryness. Four grams (4 g) of the crude hexane extract (oil) was dissolved in methylene chloride and impregnated with 4.0 g of Celite then concentrated to dryness over a rotary evaporator. The powder was chromatographed over 20 g, 60 mesh silica gel column of size 50 cm long 1.5 cm diameter. The column was eluted with hexane-EtOAc (starting from 5–70 %), then with methanol. Eighty (50 mL) fractions were collected, concentrated and grouped based on TLC profile. The fractions were combined based on comparative TLC profiles. Fractions 1–4 were combined and washed with acetone and to afford a 48 mg white paste designated AMJ1, R_f_ = 0.52, TLC (Hex/EtOAc) 1:9).

Major compound (**1**) of AMJ1, white oil: ^13^C NMR (CDCl_3_, 125 MHz): *δ* 204.1 (C-2), 169.9 (C-4), 121.4 (C-3), 57.0 (C-10), 56.5 (C-1), 56.0 (C-5), 54.5 (C-6), 45.4 (C-7), 36.7 (C-9), 31.8 (C-11), 23.7 (C-15), 22.0 (C-8), 20.4 (C-14), 20.0 (C-13), 19.5 (C-12).

### Treatment of Second Portion of Crude Hexane Extract of *C. articulatus*

Ten and half grams (10.5 g) of the crude hexane extract was treated similarly as mention above with methylene chloride then impregnation was done with 11.0 g of Celite and concentrated to dryness over a rotary evaporator. The powder was chromatographed over 53 g, 60 mesh silica gel column of size 50 cm long 4 cm diameter. The column was eluted successively with pure hexane while increasing the polarity with EtOAc. Sixty (50 mL) fractions were collected, concentrated and grouped based on TLC profile.

Fractions 1–5 were combined and subjected to gel permeation via Sephadex LH-20 (CH_2_Cl_2_) to afford a white oil designated 79 mg of AMJ2, R_f_ = 0.54 TLC (Hex/EtOAc) 2:8).

Compound (**2**): ^13^C NMR (CDCl_3_, 125 MHz): *δ* 180.4 (C-1), 130.1 (C-13), 130.0 (C-9), 128.0 (C-12, C-10), 34.3 (C-2), 31.6 (C-16), 29.8 (C-7), 29.6 (C-15), 29.4 (C-6), 29.2 (C-4, C-5), 27.3 (C-8, C-14), 25.7 (C-11), 25.0 (C-3), 22.9 (C-17), 14.2 (C-18).

### Isolation of *O. ochengi* Adult Worms

The isolation of *O. ochengi* adult worms was done as described previously [15]. The duration from the slaughtering of a cow to the harvesting of parasites from the skin was always less than 2 h to avoid bacterial contamination. Briefly, fresh pieces of umbilical cattle skin with palpable nodules bought from local slaughterhouses were washed, drained and sterilized with 70 % ethanol. *O. ochengi* adult worms were carefully scraped out of the nodules as single masses and temporarily submerged in 1 mL complete culture medium, CCM [RPMI-1640 (SIGMA, USA) supplemented with 25 mM HEPES, 2 g/L sodium bicarbonate, 2 mM l-glutamine, 5 % new born calf serum (SIGMA, USA), 150 units/mL penicillin, 150 μg/mL streptomycin and 0.5 μg/mL amphotericin B (SIGMA, USA), pH 7.4)] using 24-well plates. The adult worms were allowed in the culture medium overnight in a CO_2_ incubator, during which period the male worms migrated out of the nodular masses. Only wells containing viable worms received treatment with test metabolite. Damaged worms and worms from putrefied nodules were discarded. The viability of worms retained for the assay was ascertained by visual and microscopic examination of adult male worm motility using an inverted microscope.

### Isolation of *O. ochengi* Microfilariae

The cattle skin was obtained as described for adult worms. About 5 skin snips were obtained from different locations of the skin and incubated separately in small amounts of CCM for 30 min. Emerged Mfs were qualified and quantified for *O. ochengi* species with the aid of an inverted microscope. A selected piece of skin, rich in *O. ochengi* Mfs was carefully shaved with a razor blade, and then rinsed with distilled water. It was dabbed with a clean tea cloth to eliminate excess moisture and covered entirely with 70 % ethanol. The latter was allowed to evaporate completely in a horizontal flow sterile hood. The ethanol treatment was repeated once. The sterilized skin was tautly attached onto an autoclaved, cylindrical piece of wood using autoclaved thumb nails and close (about 1 mm apart) criss-cross cuts were made into the epidermis and dermis. The assembly was incubated in the culture medium for 4–6 h. The emerged and highly motile *O. ochengi* microfilariae were concentrated by centrifugation at 400×*g* for 10 min and then quantified.

### Preparation of Mammalian Cells

Monkey kidney epithelial cells (LLC-MK2) (ATCC, USA) were cultured at 37 °C in humidified air with 5 % CO_2_ in a HeraCell-150 incubator (Thermo Electron, Germany) until the cell layer is almost confluent. The cells were rinsed with a solution of 0.125 % trypsin and 0.5 mM EDTA in medium 199 (Sigma, USA) and kept in the same mixture for less than 1 h for them to be dislodged. The cell suspension was centrifuged at 560×*g* for 10 min, the supernatant discarded and the pellet re-suspended to 2 × 10^5^ cells/mL in CCM. The cell suspension was dispensed into 96-well microtitre plates (200 μL/well) and kept in the incubator for 3–5 days for cells to grow and become fully confluent. These cells served as feeder layer for the Mfs assays and were also used for cytotoxicity studies.

### Preparation of Stock Solutions of Isolated Metabolites

Twenty-five milligrams (25 mg) of each compound was weighed and dissolved in microtubes containing 1 mL of 99.9 % pure dimethyl sulfoxide (DMSO) (SIGMA, USA) to obtain stock solutions of 25 mg/mL. Complete dissolution was achieved by vortexing. The solutions were stored at −20 °C before they were used in the assays.

### Anti-filarial Screening of Plant-Derived Metabolites

#### Primary Screens on Adult Worms

This was done to eliminate inactive metabolites. Adult worm assays were conducted in 24-well plates (NUNC, USA) at 37 °C in humidified air containing 5 % CO_2_ for 5 days (120 h) without change of medium. Nodular masses (each generally containing a few males and a female worm) were first put in the wells (with 1 mL CCM) without extract overnight to confirm their viability and for adult males to migrate from the nodule into the culture medium. 1 mL of CCM containing 1000 μg/mL of extract was then added into each of quadruplicate wells to give a single final concentration of 500 μg/mL. Four nodular masses each, were used in the negative control (2 % DMSO in CCM only) and in the positive control (10 µM NYBC01, a gold conjugated compound) wells in which each well also received only one nodular mass. After 5 days incubation adult male viability was assessed based on motility scores using an inverted microscope. Motility score was on a scale of 4 (vigorous or normal movement of whole worm, corresponding to 0 % inhibition of worm motility), 3 (near normal movement of whole worm or 25 % inhibition of worm motility), 2 (whole body of worm motile but sluggish i.e. 50 % inhibition of worm motility), 1 (only head or tail of worm moving i.e. 75 % inhibition of worm motility), 0 (completely immotile worm i.e. 100 % inhibition of worm motility). A metabolite was considered active if there was a 100 % inhibition of adult male worm motility; or moderately active for a motility inhibition of 50–99 %; and inactive if the inhibition was less than 50 %.

Adult female worm viability was assessed by the MTT/formazan assay in which each nodular mass was placed in a well of a 48-well microtitre plate containing 500 μL/well of 0.5 mg/mL MTT (Sigma, USA) in incomplete RPMI culture medium, and then incubated in the dark at 37 °C for 30 min. Adult female worm viability was taken as mean % inhibition of formazan formation relative to negative control at 120 h post addition of plant extract. An extract was considered active on the adult female worm if there was a 90 % or greater inhibition of formazan formation compared to the negative controls; or moderately active if the inhibition was 50–89 %. It was considered inactive if the inhibition was less than 50 %. Adult worm death positively correlates with inhibition of formazan formation. Inhibition of formazan formation was assessed by visually observing the worms after incubation in MTT or by colorimetry. Each nodular mass was placed in 500 μL of DMSO in a 48-well plate to allow the colour to elute from the worm for 1 h. After shaking the plate, 200 μL of the coloured formazan solution from each well was pipetted into the wells of a microtitre plate (78 wells) and the optical density read at 490 nm. Adult female worm viability was taken as mean % inhibition of formazan formation relative to negative control at 120 h post addition of the test compound.

$$ {\text{Percent inhibition of formazan formation}} = \frac{{{\text{OD of negative control}} - {\text{OD of treatment}}}}{\text{OD of negative control}} \times 100 $$

#### Primary Screen on Microfilariae

The isolated metabolites were also tested on Mfs at a single concentration of 500 μg/mL, in triplicate wells. The Mfs assay was conducted in 96-well microtitre plates (15 mfs in 200 μL CCM per well) at 37 °C in humidified air containing 5 % CO_2_ for 5 days without any change of medium. Fully confluent monkey kidney epithelial cells, serving as feeder layer, were co-cultured with the Mfs. The medium used in preparing the feeder cell layer was removed by a swift decantation before fresh CCM containing plant test metabolite (100 μL) and worms (100 μL) were immediately added. Ivermectin (20 μg/mL) and 2 % DMSO served as the positive and negative controls respectively. Mfs motility scores (viability) were done on a scale of 0 (immotile), through 0.25 (only tail or head shaking occasionally), through 0.5 (whole body motile, but sluggishly or with difficulties), to 1 (almost vigorous to vigorous motility). Scores were made every 24 h, terminating at 120 h using an inverted microscope. Any culture with microbial contamination was not considered. Mfs viability was taken as the mean % reduction at 120 h (day 5) after addition of drug. A metabolite was considered active if there was a 100 % reduction in mfs motility; or moderately active for a motility reduction of 50–99 %; and inactive if the reduction was less than 50 %.

#### Secondary Screens on Microfilariae and Adult Worms

This was done to confirm the activity of the metabolites that showed filaricidal activity in the primary screen, and to determine their IC_50_, IC_100_ and selectivity index (SI) values. The metabolites were retested as described under primary screens at serial dilutions from 500 to 7.81 μg/mL using 24-well plates for adult worms and 96-well plates for Mfs. All assays were repeated at least three times and the results obtained are the mean values at each concentration. The graphical analysis was done using Microsoft Excel 2010 (Microsoft Corporation, USA). IC_50_ determination was done using GraphPad Prism software (version 6).

### Toxicity Studies

#### Cytotoxicity Studies

This was done as part of the Mfs assay on the active compounds through observations on the monkey kidney epithelial cells on day 5. An examination of the deformities and degree of detachment of the monkey kidney cells was done. Dead or deformed cells were usually detached from the bottom of the vessel and were rounded in shape. The IC_50_ values for these mammalian cells were determined graphically using data from microscopy. The selectivity index (SI) values were calculated using the ratio:

$$ {\text{SI}} = {\text{IC}}_{ 50}\;{\text{of drug on mammalian cell}}/{\text{IC}}_{ 50}\;{\text{of drug on parasite }}\left( {\text{Mfs}} \right) $$

#### In Silico “Drug-Likeness” of Active Isolated Compounds

The “drug-likeness” of the isolated compounds was assessed using Lipinski criteria [9], from computed molecular properties of the geometry optimized structures. All 3D molecular structures of the compounds were generated using the graphical user interface (GUI) of the MOE software [16] running on a Linux workstation with a 3.5 GHz Intel Core2 Duo processor, and energy minimization was subsequently carried out using the AM1 semiempirical approach implemented in MOPAC24 [17] until a gradient of 0.001 kcal/mol was reached. The 3D structures generated were then saved as .mol2 files subsequently included into a MOE database (.mdb). The MW, NRB, lipophilicity parameter (log P), HBA, HBD and Lipinski violations were calculated using the molecular descriptor calculator included in the QuSAR module of the MOE package [16].

## Conclusions

AMJ1 [containing mustakone (**1**) as major component] and linoleic acid (**2**) have been isolated from the roots/rhizomes of *Cyperus articulatus*. Our investigations show that both phytochemicals were able to kill both the microfilariae and adult worms of *O. ochengi* in a dose dependent manner. This study therefore provides the first ever evidence of the anti-onchcerca efficacy of AJM1-containing mustakone and linoleic acid. Efforts are being made to identify the actual compound imparting high activity to AMJ1. Thus these metabolites may provide a lead for design and development of new antifilarial agents.

## Electronic supplementary material

Below is the link to the electronic supplementary material.

Supplementary material 1 (DOCX 1470 kb)
