# Supplementary material for: Anti-onchocerca Metabolites from Cyperus articulatus: Isolation, In Vitro Activity and In Silico ‘Drug-Likeness’
Source: Nat Prod Bioprospect. 2014 Jun 10;4(4):243–9. doi: 10.1007/s13659-014-0023-5 (PMC4111868; doi:10.1007/s13659-014-0023-5)
Supplement: Supplementary file 1 — Supplementary material 1 (DOCX 1470 kb) [file 13659_2014_23_MOESM1_ESM.docx]

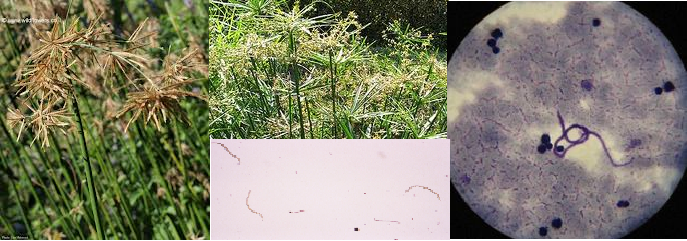


**Anti-onchocerca compounds from *Cyperus articulatus*: isolation, *in vitro* activity and *in silico* ‘drug-likeness’.**

Jonathan Alunge Metuge^1^, Smith B. Babiaka^2, 3^, James A. Mbah^2^, Fidele Ntie-Kang^2,3*^, Godfred A. Ayimele^2^ and Fidelis Cho-Ngwa^1^*

^1^ANDI Centre of Excellence, Department of Biochemistry and Molecular Biology, Faculty of Science, University of Buea, P. O. Box 63, Buea, Cameroon.

^2^Department of Chemistry, Faculty of Science, University of Buea, P. O. Box 63, Buea, Cameroon.

^3^Chemical and Bioactivity Information Centre, Chemistry Department, Faculty of Science, P. O. Box 63, Buea, University of Buea,

* Corresponding authors: [chongwa_ub@yahoo.co.uk](mailto:chongwa_ub@yahoo.co.uk) (FCN) and [ntiekfidele@gmail.com](mailto:ntiekfidele@gmail.com) (FNK)

Department of Biochemistry and Molecular Biology, Faculty of Science, University of Buea, P.O. Box 63, Buea,Cameroon

ELECTRONIC SUPPLEMENTARY MATERIALS

.

Figure S1. ESI-MS of AMJ1

**Table S1: Comparison of NMR data of AMJ1 with those of mustakone**

| **N0** | **^1^H-NMR of Mustakone**  **δ_H_(ppm*)* multiplicity, J(Hz)** | **^13^C-NMR of Mustakone** | **^1^H-NMR**  **of AMJ1** | **^13^C-NMR of AMJ1** |
| --- | --- | --- | --- | --- |
| 1 | 2.65 (1H,dd, 6.7;1.3) | 56.6 | 2.66 (1H,dd, 6.7;1.3) | 56.5 |
| 2 |  | 203.5 |  | 204.1 |
| 3 | 5.71(1H, ddq,0.9;1.3;1.4) | 121.4 | 5.71(1H, ddq,0.9;1.3;1.4) | 121.4 |
| 4 |  | 169.5 |  | 169.9 |
| 5 | 1.96 (1H,dd,6.7;0.9) | 55.9 | 1.97 (1H,dd,6.7;0.9) | 56.0 |
| 6 | 2.64 (1H,s) | 54.6 | 2.61 (1H,s) | 54.5 |
| 7 | 1.70 (1H,m) | 45.4 | 1.72 (1H,m) | 45.4 |
| 8a | 1.50 (1H,ddd, 2.8;10.5;10.5) | 21.9. | 1.50 (1H,ddd, 2.8;10.5;10.5) | 22.0 (C-8) |
| 8e | 1.70 (1H,m) | 21.9 | 1.70 (1H,m) | 22.0 (C-8) |
| 9a | 1.85 (1H,m) | 36.7 | 1.85 (1H,m) | 36.7 (C-9) |
| 9e | 1.70 (1H,m) | 36.7 | 1.70 (1H,m) | 36.7 (C-9) |
| 10 |  | 57.0 |  | 57.0 |
| 11 | 1.48 (1H,dd,6.5;6.5) | 31.7 | 1.49 | 31.8 |
| 12 | 0.83 (3H,d,6.5) | 19.4 | 0.84 | 19.5 |
| 13 | 0.81 (3H,d,6.5) | 19.9 | 0.80 (3H,d,6.5) | 19.9 |
| 14 | 1.98 (3H,d,1.4) | 20.2 | 1.97 (3H,d,1.4) | 20.3 |
| 15 | 0.94 (3H,s) | 23.5 | 0.95 | 23.7 |

**Table S2: Comparison of NMR data of AMJ2 with those of Linoleic acid in δ_H_ (ppm*)***

| **N0** | **^1^H-NMR of Linoleic acid** | **^13^C-NMR of Linoleic acid** | **^1^H-NMR**  **of AMJ2** | **^13^C-NMR of AMJ2** |
| --- | --- | --- | --- | --- |
| 1 |  | 180.5 |  | 180.4 |
| 2 | 2.34 t | 34.3 | 2.34 t | 34.3 |
| 3 | 1.59 m | 25.0 | 1.59 m | 25.0 |
| 4 | 1.25 m | 29.2 | 1.25 m | 29.2 |
| 5 | 1.25 m | 29.2 | 1.25 m | 29.2 |
| 6 | 1.25 m | 29.4 | 1.25 m | 29.4 |
| 7 | 1.25 m | 29.8 | 1.25 m | 29.8 |
| 8 | 2.00 m | 27.3 | 2.00 m | 27.3 |
| 9 | 5.34 m | 130.0 | 5.34 m | 130.0 |
| 10 | 5.34 m | 128.1 | 5.34 m | 128.1 |
| 11 | 2.80 m | 25.7 | 2.80 m | 25.7 |
| 12 | 5.34 t | 128.0 | 5.34 t | 128.0 |
| 13 | 5.34 m | 130.1 | 5.34 m | 130.1 |
| 14 | 2.00 m | 27.3 | 2.00 m | 27.3 |
| 15 | 1.25 m | 29.6 | 1.25 m | 29.6 |
| 16 | 1.25 m | 31.6 | 1.25 m | 31.6 |
| 17 | 1.25 m | 22.9 | 1.25 m | 22.9 |
| 18 | 0.89 t | 14.2 | 0.89 t | 14.2 |

^1^H and ^13^C NMR spectra

AMJ1

Linoleic acid
